# Yes-associated protein-1 overexpression in ocular surface squamous neoplasia; a potential diagnostic marker and therapeutic target

**DOI:** 10.3389/fonc.2023.1213426

**Published:** 2023-07-05

**Authors:** Peter Julius, Stepfanie N. Siyumbwa, Fred Maate, Phyllis Moonga, Guobin Kang, Trevor Kaile, John T. West, Charles Wood, Peter C. Angeletti

**Affiliations:** ^1^Department of Pathology and Microbiology, School of Medicine, University of Zambia, Lusaka, Zambia; ^2^University Teaching Hospital, Eye Hospital, Lusaka, Zambia; ^3^Department of Interdisciplinary Oncology, Louisiana State University Health Science Center, New Orleans, LA, United States; ^4^Nebraska Center for Virology and the School of Biological Sciences, University of Nebraska-Lincoln, NE, United States

**Keywords:** yes-associated protein-1 (YAP-1), ocular surface squamous neoplasia (OSSN), preinvasive OSSN, invasive OSSN, Zambia

## Abstract

Yes-associated protein-1 (YAP-1) is a Hippo system transcription factor, which serves as an oncogene in squamous cell carcinoma, and several solid tumors when the Hippo pathway is dysregulated. Yet, the activity of YAP-1 in ocular surface squamous neoplasia (OSSN) has not been determined. Here, we investigate the relationship between YAP-1 overexpression and OSSN. Using a cross-sectional study design, we recruited 227 OSSN patients from the University Teaching Hospitals in Lusaka, Zambia. Immunohistochemistry was used to assess YAP-1 protein overexpression in tumor tissue relative to surrounding benign squamous epithelium. OSSN patient samples (preinvasive, n = 62, 27% and invasive, n = 165, 73%) were studied. One hundred forty-nine invasive tumors contained adjacent preinvasive tissue, bringing the total number of preinvasive lesions examined to 211 (62 + 149). There was adjacent benign squamous epithelium in 50.2% (114/227) of OSSN samples. Nuclear YAP- 1 was significantly overexpressed in preinvasive (Fisher’s (F): p <.0001, Monte Carlo (MC): p <.0001) and invasive (F: p <.0001, MC: p <.0001) OSSN in comparison to adjacent benign squamous epithelium when analyzed for basal keratinocyte positive count, staining intensity, expression pattern, and Immunostaining intensity-distribution index. YAP-1 expression did not differ between preinvasive and invasive OSSN (p >.05), keratinizing and non- keratinizing cancer (p >.05), or between T1/T2 and T3/T4 stages in invasive tumors (p >.05). However, grade 2 and 3 tumors had significantly stronger nucleus YAP-1 overexpression intensity than grade 1 tumors (F: p = .0078, MC: p = .0489). By immunohistochemistry, we identified significant overexpression (upregulation of YAP-1 protein expression) in preinvasive and invasive OSSN lesions compared to neighboring benign squamous epithelium. YAP-1 expression was significantly higher in poorly and moderately differentiated invasive squamous cancer than in well-differentiated carcinomas. Overexpression of YAP-1 within the margin of preinvasive and invasive OSSN, but not in the neighboring normal epithelium, indicates that it plays a role in the development and progression of OSSN.

## Introduction

Ocular surface squamous neoplasia (OSSN) includes preinvasive and invasive squamous cancers affecting the ocular surface (1, 2). If left untreated, OSSN can lead to severe complications, including loss of vision, disfigurement following disease progression or surgical treatment, loss of an eye, metastasis, or death (1, 2). Currently, topical 5-fluorouracil (5-FU) or Mitomycin C, with or without surgical excision, is used to treat OSSN in its early stages (1, 3, 4).

The Hippo pathway functions in cell signaling and is critical in regulating organ growth, cell proliferation, and cell death *via* apoptosis (5, 6). Dysregulation of the Hippo pathway, including the overexpression or loss of the transcriptional co-activator Yes-associated protein 1 (YAP-1), has been linked to the development of cancer (7, 8). YAP-1 is a transcriptional co-activator and negative regulator of the Hippo pathway (9). Its overexpression or loss is associated with cancer development, and it promotes malignant transformation, the proliferation of cancerous stem cells, and drug resistance (10). YAP-1 is a transcription co-activator (9) regulated by the Hippo pathway. The fundamental mechanism by which the Hippo pathway suppresses the growth of tumors is *via* the reduction of YAP- 1 transcriptional activity. The Hippo pathway also drives YAP-1 phosphorylation. The interaction between phosphorylated YAP-1 and 14-3-3 protein keeps YAP-1 in the cytoplasm, targeting it for ubiquitin-mediated proteasomal degradation. YAP-1 translocates to the nucleus when the Hippo pathway is inactive, where it binds TEAD. The YAP-TEAD complex stimulates the transcription of cell proliferation-related genes, such as AMOTL2, AREG, BIRC5, CTGF, and CYR61, which contribute to the initiation, proliferation, survival, progression, invasion, and metastasis of cancer (11).

YAP-1 protein is overexpressed in various cancers, including colorectal cancer, gastric cancer, hepatocellular carcinoma, breast cancer, hedgehog-associated medulloblastoma, esophageal and oral squamous cell carcinoma (SCC), leukemia and other solid tumors (7, 8, 12–35), and has been referred to as an oncogene.

Overexpression is often associated with a poor prognosis, progression, tissue invasion, recurrence, poor tumor differentiation, metastasis, poor disease-free survival, and worse overall survival. For example, YAP-1 overexpression in colorectal cancer is associated with advanced pTNM stage, positive nodal status, tumor status, and cyclin D1 overexpression (36). Similarly, Xia (11) found that YAP-1 expression is associated with poor survival in patients with ovarian cancer and that high YAP-1 expression levels positively correlate with TEAD4 gene expression levels. YAP-1 contributes to the multidrug resistance of lung and esophageal small-cell carcinoma (28, 29). Normal YAP-1 expression in epithelial tissue is observed as weak cytoplasmic staining limited to the basal-parabasal epithelial layer with occasional scattered nucleus staining (37, 38). In contrast, overexpression is observed as a strong block of staining involving both the nucleus and cytoplasm (37, 38). Complete loss of nucleus and cytoplasmic staining is interpreted as loss of expression. While most solid tumors are associated with overexpression of YAP-1, loss of expression is seen in and defines neuroendocrine differentiation of lung cancer (16). Compared to YAP-1-positive groups, Barry (14) found that YAP-1 deficiency was associated with high-grade, stage IV illness and a lower likelihood of patient survival. The studies led to the conclusion that YAP-1 could independently inhibit Wnt signaling. This suggests a central role for YAP-1 in cancer, and thus, it is essential to elucidate how it functions, particularly in response to viral oncogene expression. Several studies have implicated a variety of tumor virus oncogenes in affecting the hippo pathway by modulation of YAP-1 expression. For example, Human papillomaviruses (HPVs), Polyomaviruses (PyV), Kaposi’s sarcoma-associated herpesvirus (KSHV) and Adenovirus have been reported to impact YAP-1 expression (39–44). Other studies have found that EBV LMP-1 expression leads to nuclear accumulation of YAP-1 in transformed epithelia by degrading its partner protein, the transcriptional co-activator with PDZ-binding motif (TAZ) (45). These previous studies point to the potential utility of YAP-1 as a biomarker for several virally induced cancers.

Emerging evidence suggests that YAP-1 may contribute to developing resistance to targeted therapies and chemotherapy drugs, including 5-FU, a medicine commonly used to treat OSSN. 5-FU resistance is associated with YAP-1 overexpression in gastric and colorectal cancer (46–48). Inhibiting YAP-1 has been shown to reduce cancer cell growth and proliferation, making it a potential target for treating YAP-associated diseases (10, 23, 33). While YAP-1 staining and its role in diagnosis and prognosis has been studied in most solid tumors, it has not been investigated in OSSN, and its expression and potential role in 5-FU resistance in OSSN are still unknown. More research is needed to understand the role of YAP-1 in OSSN and to determine whether YAP-1 has the potential as a biomarker or therapeutic target for this disease. In this study, we investigate the expression of YAP-1 in preinvasive and invasive OSSN compared to the adjacent non-dysplastic epithelium to determine whether it is a potential diagnostic and therapeutic target.

## Methods

### Study design

This exploratory cross-sectional study enrolled 227 individuals with histologically confirmed OSSN. Between November 2017 and September 2022, all participants were recruited from the University Teaching Hospitals (UTH), Eye Hospital, as detailed previously (49, 50). Ethical approval was granted by the University of Zambia Biomedical Research Ethics Committee (IRB # 015-05-17), the Zambia National Health Research Authority, the University of Nebraska-Institutional Lincoln’s Review Board (IRB # 20170817442FB), and the Louisiana State University Institutional Review Board (IRB # 2252). Patients diagnosed with preinvasive and invasive OSSN and with sufficient formalin-fixed paraffin-embedded (FFPE) tissue for immunohistochemistry (IHC) were included in this investigation.

### Histopathology

Each lesion was evaluated for invasive SCC with its histologic type, preinvasive lesion with its grade, and surrounding normal (non-dysplastic) epithelium. Of the 227 patients diagnosed with OSSN in this study, 62 lesions were preinvasive, and 165 were invasive. Approximately 90.3% (n = 149) of invasive OSSN tumor samples contained a preinvasive component adjacent to the invasive carcinoma. This allowed 211 preinvasive OSSN patient samples for YAP-1 evaluation (including 62 preinvasive OSSN). A normal ocular surface squamous epithelium was observed in 114 of 227 (50.2%) histologic samples adjacent to a dysplastic or invasive OSSN.

### Sociodemographic and clinical information

As explained in our previous papers, each participant’s sociodemographic information, clinical evaluation data, and routine laboratory examinations were collected (49, 50). The expression of latent Epstein-Barr virus nuclear antigen 1 (EBNA1) and CDKN2A/p16INK4A (p16) for the detection of Epstein-Barr virus and Human papillomavirus infection from our earlier publication (50)was 88.7% and 4.9%, respectively for the research participants included in the study.

Age, gender, HIV status, ART history, CD4 count, plasma viral load, diagnostic category, tumor subtype, grade, and stage information were gathered. All individuals were diagnosed with OSSN that had not been treated previously.

The clinical staging of the tumors was conducted per the 8th edition of the American Joint Committee on Cancer Staging handbook (2018) (51). After a clinical evaluation, tumor samples were collected and processed for histologic confirmation of the OSSN diagnosis and IHC, as previously reported. Two pathologists reviewed and assessed Hematoxylin and Eosin (H & E) and Mucicarmine stained slides at multiple levels to confirm the presence of the tumor tissue on the slides before IHC. The ultimate diagnosis was reached independently, and when disagreements arose, by consensus. We divided tumors into preinvasive (cornea/conjunctiva intraepithelial neoplasia) and invasive (cornea-conjunctiva SCC) OSSN based on the 4^th^ edition of the World Health Organization (WHO) classification of eye cancers (52). Preinvasive tumors were classified as cornea-conjunctiva intraepithelial neoplasia (CIN) types 1, 2, and 3 (CIN 1, CIN 2, and CIN 3) and carcinoma-*in-situ* (CIS). According to the WHO Classification of eye cancers, 4th edition (52). Subtypes and grades were assigned to invasive OSSN. Every invasive tumor was evaluated for vascular and perineural invasion. Each subject was assessed for lymph node enlargement and distant metastases at the time of recruitment.

### Immunohistochemistry

Using primary antibody: Rabbit monoclonal anti-YAP-1 antibody (pSer127, Cat. # PA5-17481, 100 L, Thermo Scientific. Rockford, IL61105, USA) at a 1:200 dilution, the expression of YAP-1 was analyzed by IHC on FFPE tissue sections. Briefly, 6-micron-thick tissue sections were cut from tissue blocks and placed on charged slides. Overnight, the slides were incubated at 60°C. Tissue sections were deparaffinized in two 5-minute xylene washes and rehydrated in five-minute ethanol washes of 100%, 100%, 85%, and 70% concentrations. A 3% hydrogen peroxide (H2O2) methanol solution was used at room temperature for 30 minutes to inhibit endogenous peroxidase activity. Slides were washed in distilled water (three changes at three minutes each). Antigen retrieval was carried out by heating the samples for 15 minutes in a steamer with a 10 mM citrate buffer at a pH of 6. Twenty minutes were given for the slides to cool in the buffer. A 1X phosphate buffered saline (PBS buffer) was used to rinse the tissue. In a humidity chamber, slides were incubated with blocking solution, normal goat serum (10%), for 30 minutes at room temperature. Primary antibody-coated slides were incubated overnight at 4°C in a humidity chamber. The slides were then allowed to warm to room temperature for one hour before being rinsed in 1X PBS (three changes at three minutes each). Anti-Rabbit HRP Labeled secondary antibody (K4001, Dako, USA) was then incubated at room temperature for 30 minutes with tissue slides. Slides were washed in 1X PBS (three changes at three minutes each). The signal was produced with the diaminobenzidine (DAB) substrate-Chromogen System (K3468, Dako, USA). As a counterstain, Harris hematoxylin was used.

The stained slides were digitized at X40 magnification using a slides scanner (MoticEasyScan Pro 6, Motic, USA) and analyzed using the Motic digital slides assistant software (Motic DSAssistant VM 3.0). A faint cytoplasmic stain with or scattered nuclear staining restricted to basal keratinocytes characterized normal YAP-1 staining. The complete absence of YAP-1 staining was regarded as negative, while nuclear staining with or without substantial cytoplasmic staining indicated YAP-1 overexpression. As detailed earlier (37, 53), each field was evaluated as follows: Nuclear staining intensity was assessed as follows: 0: absence (colorless), 1: mild (light yellow), 2: moderate (yellowish brown), and 3: intense (chocolate brown). Basal keratinocyte nuclear staining count was scored as follows: 0 =< 5%; 1 = 5%–25%; 2 = 26%–50%; 3 = 51%–75%; 4 = > 75% stained cells. The Immunostaining intensity-distribution index (IIDI)was calculated by multiplying, for each field, the score for the percentage of positively stained cells by the score for that field’s staining intensity. The score was broken down as follows: negative = 0, weakly positive = 1–4, moderately positive = 6–8, and strong positive = 9–12.

### Statistical analysis

IBM SPSS Statistics 26 was used for data cleaning. The tables were generated using R Statistical language (version 4.2.2; R Core Team, 2022) using gtsummary and flextable (other functions used are given in the **Supplementary material**: R code). The status of YAP-1 was expressed as frequency/total (percentage). The *IHC YAP-1 status* was studied as the dependent variable, and its association with independent variables was evaluated. The Chi-square and Fisher’s exact probability tests (37, 54) were employed to analyze the relationship between YAP-1 IHC reactivity and histopathologic factors, with all assumptions considered. SAS OnDemand was used to find these associations.

Monte Carlo simulation validated Fisher’s exact test results by generating many random samples to compare the results to the observed data (Code, Number of samples=10,000 samples, and SEED=5678 numbers are given in the **Supplementary material**).

## Results

### Patient and samples results

Our study included tumor samples from 227 patients with histologically confirmed OSSN (**Table 1**). Females (60.8%) and HIV-positive subjects (70.5%) dominated the study. The participants were young, with a median age of 38 years (Range: 19-76 years). Most (n = 130/160, 81%) of HIV-positive participants knew their status before the OSSN diagnosis and received antiretroviral therapy, while 18.8% were newly diagnosed with HIV. The median length of HIV infection was 2.0 years (range: 0.0-31.0 years), while the median duration of ART use was 2.0 years (range: 0.0-20.0 years. At recruitment, 90% of the HIV+ participants had blood samples evaluated for CD4 count.

**Table 1:** Sociodemographic and Clinicopathological characteristics of the participants and the tumors.

Variable		F/T (%); Median [IQR] Range; Mean ± SD
**Age**	Male	38 [31-45] 19-76 yrs. 89/227 (39.2)
	Female	138/227 (60.8)
**HIV status**	Positive	160/227 (70.5)
	Negative	67/227 (29.5)
**HIV long** **CD4 Count among HIV Positive**		2.0 (0.6-5.0) 0.0 - 31.0 years* 210.0 [116.0 – 404.0] 4.0 - 1383.0 cells/mm^3^
**CD4 Count [Category (C)]**	<200	69/144 (47.9)
**Plasma Viral load and CD4 Count**	>200	75/144 (52.1) 30 [0.0 – 3.992.0] 0.0 – 2.0 x 10^6^ copies/mL
**Plasma Viral load (C)**	<200	54/84 (64.3)
	>200	30/84 (35.7)
**ART Ever and Duration**	Yes	130/160 (81.2) 2.0 (0.6 -5.0) 0.0 - 20 years*
**Diagnosis**	Preinvasive OSSN	62/227 (27.3)
	Invasive OSSN	165/227 (72.7)
**Preinvasive tumor grade**	CIN-I	1/62 (1.6)
	CIN-II	6/62 (9.7)
	CIN-III	18/62 (29.0)
	CIS	37/62 (59.7)
**Preinvasive tumor grade (C)**	CIN I and II	7/62 (11.3)
	CIN III and CIS	55/62 (88.7)
**Invasive tumor subtype**	SPCC	4/165 (2.4)
	BSCC	4/165 (2.4)
	CSCC	157/165 (95.2)
**Invasive tumor grouped**	Keratinizing	157/165 (95.2)
	Non-keratinizing	8/165 (4.8)
**Grade of invasive tumor**	G1	20/165 (12.1)
	G2	133/165 (80.6)
	G3	12/165 (7.3)
**AJCC Stage**	pT1	40/165 (24.2)
	pT2	40/165 (24.2)
	pT3	83/165 (50.3)
	pT4	2/165 (1.2)
**AJCC Stage (C)**	T1/T2	80/165 (48.5)
	T3/T4	85/165 (51.5)
**P16 in Tumor**	Positive	11/224 (4.9)
**EBNA-1 in Tumor**	Positive	197/222 (88.7)

F/T (%): Frequency/total Median (IQR) Range; Mean ± SD; (C): categorized *0-9 months = 0.0 - 0.9 months & 1 = 1 year and above.

The median CD4 count was 210 (Interquartile range, IQR: 116 – 404, Range: 4 - 1383) cells/mm^3^, with 47.9% severely immunosuppressed (CD4 count < 200 cells/mm^3^). Viral load testing was conducted in 84/160 (52.5%) HIV+ participants. The median plasma viral load was 30 (IQR: 0.0 – 3,992.0, Range 0.0 – 2.0 x 10^6^) copies/ml, and most (64.3%) of HIV+ participants were virally suppressed (Viral load < 200 copies/ml).

The OSSN diagnoses included invasive (72.7%) and preinvasive (27.3%) cancers. Normal ocular surface squamous epithelium was present in 114/227 (50.2%) histologic samples adjacent to a dysplastic or invasive tumor. About 90.3% (n = 149/165) of invasive OSSN tumor samples contained a preinvasive component adjacent to the invasive carcinoma. This allowed 211 preinvasive OSSN patient samples for YAP-1 evaluation (including 62 preinvasive OSSN). On each slide, the preinvasive, invasive, and normal epithelium were evaluated for the pattern of YAP-1 staining. CIN III and CIS accounted for 88.7% (55/62) of the preinvasive OSSN cases.

Conventional (keratinizing) SCC predominated in invasive OSSN (157/165, 95.2%) cases. Other invasive carcinoma subtypes were Basaloid SCC (4/165, 2.4%) and Spindle cell carcinoma (4/165, 2.4%). Most invasive tumors (133/165, 80.6%) were moderately differentiated, whereas well and poorly-differentiated invasive squamous malignancies comprised 12.1% and 7.3% of cases, respectively. pT3 and pT4 stages comprised most malignancies (51.5%). Eight (3.5%) participants had clinically enlarged head and neck lymph nodes. Only one of the eight patients with palpable lymph nodes had clinically distant metastases. Two hundred and twenty-two (222) of the total cases were previously evaluated for EBV infection using Epstein-Barr virus nuclear antigen-1 (EBNA-1) immunohistochemistry (IHC), and 88.7% tested EBV positive localized to the tumor cells. Similarly, 224 tumors had p16 IHC results, with 4.9% of cases showing p16 overexpression (**Table 1**).

### Immunohistochemistry results

#### Adjacent normal ocular surface epithelium

**Figure 1:**
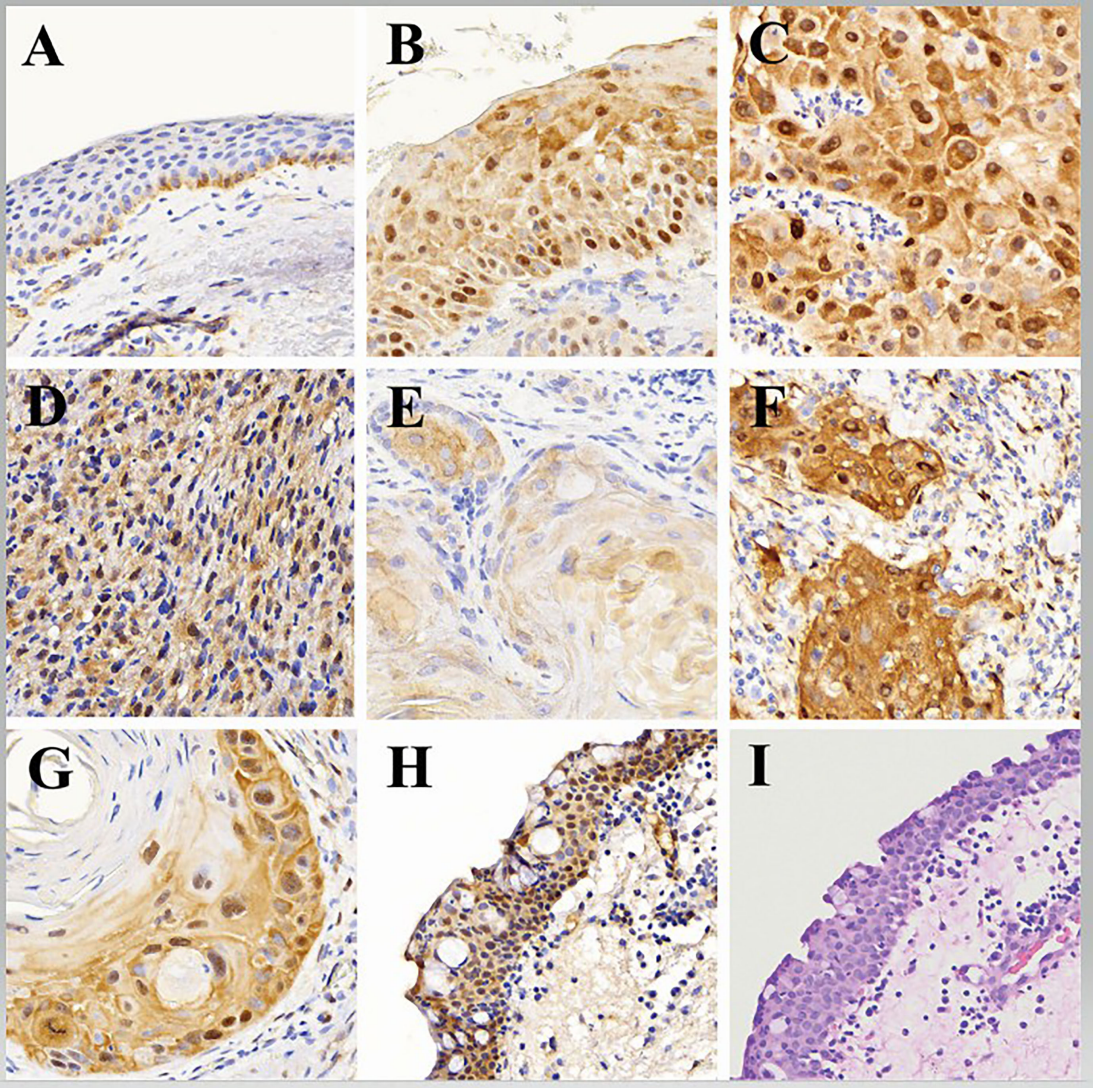
Representative histologic images showing: **(A)** adjacent normal (benign) epithelium with weak YAP-1 cytoplasmic staining and scattered weak nuclei YAP-1 staining (YAP-1-normal expression) in the basal keratinocytes, **(B)** Preinvasive ocular surface squamous neoplasia with strong block YAP-1 staining in cytoplasm and nuclei (YAP-1-overexpression) involving total thickness of the epithelium, **(C)** Invasive ocular surface squamous neoplasia with strong block YAP-1 staining in cytoplasm and nuclei (YAP-1-overexpression) involving full thickness of the epithelium, **(D)** Nonkeratinizing invasive ocular surface squamous neoplasia (spindle cell carcinoma) with strong block YAP-1 staining in cytoplasm and nuclei (YAP-1-overexpression) involving full thickness of the epithelium, **(E)** Keratinizing invasive ocular surface squamous neoplasia with weak YAP-1 staining in cytoplasm but not the nuclei (YAP-1-normal expression) involving full thickness of the epithelium, **(F)** Invasive ocular surface squamous neoplasia with strong YAP-1 staining in nuclei of spindle stromal cells but not the immune cells, **(G)** Keratinizing invasive ocular surface squamous neoplasia with YAP-1-overexpression in the basal keratinocytes and disposed in a parabasal pattern involving more than 2/3 of the epithelial thickness with weak to no expression in the central keratin pearl, **(H)** Normal adjacent epithelium with basal cell hyperplasia showing YAP-1-overexpression in the cytoplasm and nuclei involving full epithelial thickness, and, **(I)** Normal adjacent epithelium stained with hematoxylin and eosin (H&E) showing basal cell hyperplasia.

Adjacent normal conjunctiva epithelium was observed in 114 (50.2%) of the 227 OSSN cases in the study samples (**Figures 1A, H, I**). YAP-1 expression in the adjacent normal ocular surface epithelium was observed in the basal keratinocytes involving both the cytoplasm and the nucleus. YAP-1 cytoplasmic staining was uniformly weak in all but one case and restricted to the basal and parabasal layers. Nuclear staining was observed in all the cases. Most (99.1%) nuclei staining presented as a scattered pattern, involving less than 25% of the basal keratinocytes limited to the basal layer (**Figure 1A**) of the epithelium; however, one (0.9%)case showed YAP-1 overexpression with strong staining intensity involving ⅔ epithelial thickness in a parabasal pattern (**Figure 1H**). Nuclear intensity staining was uniformly weak in cases showing a YAP-1 scattered pattern (**Figure 1A**). The IIDI for the normal adjacent epithelium ranged from negative in 9 (7.9%), weak in 104 (91.2%), and strong in one (0.9%) of the cases (**Table 2**). The nuclear staining in a scattered pattern with the weak cytoplasmic staining limited to the basal layer was considered normal YAP-1 staining in the cornea and conjunctiva for our study. The tumor sample that showed YAP-1 overexpression in the normal adjacent epithelium was of a 29-year-old HIV+ female patient diagnosed with a well-differentiated invasive squamous carcinoma. The patient’s tumor contained both an invasive and a preinvasive OSSN component, and both had YAP-1 overexpression. The surrounding normal conjunctival epithelium displayed hyperplastic basal cells and elevated YAP-1 expression (**Figure 1H**).

**Table 2:** Comparison of YAP-1 overexpression between preinvasive and invasive OSSN with the adjacent normal squamous epithelium.

	Normal	Preinvasive	Invasive
**Overall**	227	227	227
Component			
Absent	113/227 (49.8)^#^	16/227 (7.0)	62/227 (27.3)
Present	114/227 (50.2)	211/227 (93.0)	165/227 (72.7)
YAP-1 staining			
Normal expression	113/114 (99.1)^#^	4/211 (1.9)	2/165 (1.2)
Overexpression	1/114 (0.9)	207/211 (98.1)	163/165 (98.8)
***N vs. P vs. I***	Fisher’s: p<.0001, MC: p <.0001		
***N vs. P***	Fisher’s: p<.0001, MC: -		
***N vs. I***	Fisher’s: p<.0001, MC: -		
***P vs. I***	Fisher’s: p= .6991, MC: -		
Localization			
Nucleus and Cytoplasm	114/114 (100.0)	211/211 (100.0)	165/165 (100.0)
Nucleus staining			
Present	114/114 (100.0)	211/211 (100.0)	165/165 (100.0)
Primary Pattern			
Parabasal diffuse overexpression	1/114 (0.9)	207/211 (98.1)	163/165 (98.8)
Basal expression	0/114 (0.0)	0/211 (0.0)	0/165 (0.0)
Scattered	113/114 (99.1)^#^	4/211 (1.9)	2/165 (1.2)
***N vs. P vs. I***	Fisher’s: p<.0001, MC: p<.0001		
***N vs. P***	Fisher’s: p<.0001, MC: p<.0001		
***N vs. I***	Fisher’s: p<.0001, MC: p<.0001		
***P vs. I***	Fisher’s: p=.6991, MC: -		
Positive count			
<5%	9/114(7.9)	0/211 (0.0)	0/165 (0.0)
5%-25%	104/114 (91.2)^#^	4/211 (1.9)	2/165 (1.2)
26%-50%	0/114 (0.0)	0/211 (0.0)	0/165 (0.0)
51%-75%	1/114 (0.9)	0/211 (0.0)	0/165 (0.0)
>75%	0/114 (0.0)	207/211 (98.1)	163/165 (98.8)
***N vs. P vs. I***	Fisher’s: p<.0001, MC: p<.0001		
***N vs. P***	Fisher’s: p<.0001, MC: p<.0001		
***N vs. I***	Fisher’s: p<.0001, MC: p<.0001		
***P vs. I***	Fisher’s: p=.6991, MC: -		
Intensity			
Uniformly weak	113/114 (99.1)^#^	4/211 (1.9)	3/165 (1.8)
Uniformly moderate	0/114 (0.0)	59/211 (28.0)	37/165 (22.4)
Uniformly strong	1/114 (0.9)	148/211 (70.1)	125/165 (75.8)
***N vs. P vs. I***	Fisher’s: p<.0001, MC: p<.0001		
***N vs. P***	Fisher’s: p<.0001, MC: p<.0001		
***N vs. I***	Fisher’s: p<.0001, MC: p<.0001		
***P vs. I***	Fisher’s: p= .0133, MC: p= .4620		
IIDI			
**0**	9/114 (7.9)	0/211 (0.0)	0/165 (0.0)
** +1**	104/114 (91.2)^#^	4/211 (1.9)	2/165 (1.2)
**+2**	0/114 (0.0)	0/211 (0.0)	0/165 (0.0)
**+3**	0/114 (0.0)	0/211 (0.0)	0/165 (0.0)
**+4**	0/114 (0.0)	0/211 (0.0)	1/165 (0.6)
**+6**	0/114 (0.0)	0/211 (0.0)	0/165 (0.0)
**+8**	0/114 (0.0)	59/211 (28.0)	37/165 (22.4)
**+9**	1/114 (0.9)	0/211 (0.0)	0/165 (0.0)
**+12**	0/114 (0.0)	148/211 (70.1)	125/165 (75.8)
***N vs. P vs. I***	Fisher’s: p<.0001, MC: p<.0001		
***N vs. P***	Fisher’s: p<.0001, MC: p<.0001		
***N vs. I***	Fisher’s: p<.0001, MC: p<.0001		
***P vs. I***	Fisher’s: p= .0130, MC: p= .3974		
IIDI (C)			
Negative (0)	9/114 (7.9)	0/211 (0.0)	0/165 (0.0)
Weak positive (1-4)	104/114 (91.2)^#^	4/211 (1.9)	3/165 (1.8)
Moderately positive (6–8)	0/114 (0.0)	59/211 (28.0)	37/165 (22.4)
Strongly Positive (9–12)	1/114 (0.9)	148/211 (70.1)	125/165 (75.8)
***N vs. P vs. I***	Fisher’s: p<.0001, MC: p<.0001		
***N vs. P***	Fisher’s: p<.0001, MC: p<.0001		
***N vs. I***	Fisher’s: p<.0001, MC: p<.0001		
***P vs. I***	Fisher’s: p= .0133, MC: p= .4620		
Parabasal extension			
Lower one third	113/114 (99.1)^#^	4/211 (1.9)	2/165 (1.2)
Up to two thirds	1/114 (0.9)	1/211 (0.5)	1/165 (0.6)
Up to three thirds	0/114 (0.0)	206/211 (97.6)	162/165 (98.2)
***N vs. P vs. I***	Fisher’s: p<.0001, MC: p<.0001		
***N vs. P***	Fisher’s: p<.0001, MC: p<.0001		
***N vs. I***	Fisher’s: p<.0001, MC: p<.0001		
***P vs. I***	Fisher’s: p= .1424, MC: p= .8540		

Frequency (%); MC (Monte Carlo); N (Normal), P (Preinvasive) I (Invasive); # Cell contributing to Chi-square.

Overexpression of YAP-1 was detected in stromal spindle cells within the tumor microenvironment (**Figure 1F**). but not in stromal cells outside of the tumor microenvironment (**Figure 1A**). or tumor-infiltrating immune cells.

#### Preinvasive OSSN lesions

The 211 preinvasive OSSN lesions (including 62 preinvasive OSSN and 149 preinvasive lesions adjacent to invasive OSSN) were evaluated for YAP-1 staining. Most preinvasive (98.1%) lesions showed YAP-1 overexpression (**Figure 1B**), while four cases (1.9%) showed normal expression (**Table 2**). Overexpression was characterized by moderate to strong nuclear and cytoplasmic staining as block staining (**Table 2**). Nuclei YAP-1 overexpression was disposed of in a parabasal diffuse pattern. It involved more than 80% of the basal keratinocyte, with a moderate to strong staining intensity and an IIDI score of 8+ to 12+ (**Table 2**). Nuclei YAP-1 overexpression parabasal extension involved more than ⅔ of the epithelial thickness in most (97.6%) cases (**Table 2**). The four preinvasive OSSN samples that showed normal YAP-1 expression had less than 25% nuclei positivity in the basal keratinocytes, displayed in a scattered pattern, and restricted to the lower one-third of the epithelial thickness. The nuclear staining intensity in these cases was weak, with an IIDI staining intensity of 1+ (weak +ve). YAP-1 was significantly overexpressed in preinvasive OSSN compared to the neighboring normal epithelium (98.1% *vs*. 0.9%, p <.0001). The parabasal pattern of YAP-1 expression was linked with preinvasive OSSN, whereas the scattered pattern was associated with normal surrounding epithelium (Fisher’s (F): p <.0001, Monte Carlo (MC): p <.0001). Weak nuclear staining was strongly related to the normal surrounding epithelium (Fisher’s: p <.0001, MC: p <.0001), whereas moderate to strong nuclear staining was observed in preinvasive OSSN. Preinvasive OSSN was significantly associated (F: p <.0001, MC: p <.0001) with a greater YAP-1 positive count in the basal keratinocytes than in the normal surrounding epithelium. A significant Fisher’s (p <.0001) and Monte Carlo (p <.0001) confirmed YAP-1 nuclear overexpression using higher IIDI scores in preinvasive OSSN compared to the neighboring normal epithelium (F: p <.0001, MC: p <.0001) (**Table 2**).

#### Invasive OSSN lesions

**Figure 2:**
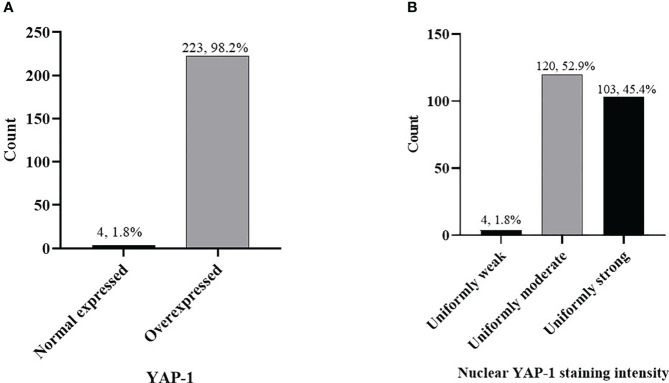
Frequency of YAP-1 expression in combined (preinvasive and invasive) OSSN **(A)** cases, YAP-1 nuclear staining intensity in combined OSSN **(B)**.

One hundred sixty-five (165) invasive OSSN lesions were analyzed and evaluated for YAP-1 staining. Most invasive (98.8%) lesions showed YAP-1 overexpression, while two cases (1.2%) showed normal expression (**Table 2**; **Figure 1C, D, F, G**). Overexpression was characterized by moderate to strong nuclear and cytoplasmic staining as block staining (**Figure 2**, **Table 2**; **Supplementary Table 1**). The nuclei YAP-1 overexpression pattern was like that seen in preinvasive OSSN. Most tumors (98.2%) had moderate to strong staining intensity and an IIDI score of 8+ to 12+. Nuclei YAP-1 overexpression parabasal extension involved more than ⅔ of the epithelial thickness in most (98.8%) cases (**Table 2**). The two invasive OSSN samples that showed normal YAP-1 expression had less than 25% nuclei positivity in the basal keratinocytes, displayed in a scattered pattern, and restricted to the lower one-third of the epithelial thickness (**Figure 1E**). The nuclear staining intensity in these cases was weak, with an IIDI staining intensity of 1+ (weak +ve). Comparison of YAP-1 expression between invasive OSSN, preinvasive OSSN, and normal adjacent epithelium (**Table 2**) revealed: that normal adjacent epithelium was significantly associated with normal YAP-1 expression, whereas the OSSN groups were observed to have YAP-1 overexpression (F: p <.0001, MC: p <.0001). We found no difference between invasive and preinvasive OSSN in YAP-1 expression (p = .6991), YAP-1 expression pattern (p = .6991), or basal keratinocyte nuclei positive counts (p = .6991). In invasive OSSN, YAP-1 staining intensity was shown to be substantially stronger (F; p = .0133) than in preinvasive OSSN; however, Monte Carlo simulation abolished the observed difference (p = .4620). These findings were confirmed using grouped and ungrouped IIDI scores (**Table 2**). In the OSSN groups, the parabasal extension of YAP-1 nuclei positive was similar (Fisher’s: p = .1424; MC: p = .8540). When we evaluated YAP-1 expression between invasive OSSN and adjacent normal epithelium, we found comparable results to what was observed with preinvasive OSSN *vs*. adjacent normal epithelium (**Table 2**). All metrics showed a significant association between YAP-1 overexpression and invasive OSSN.

Using Monte Carlo simulation to correct grouped IIDI values comparing YAP-1 expression between invasive and preinvasive OSSN, it was determined that YAP-1 was equally (p = .1275) expressed. Likewise, YAP- 1 IIDI scores did not differ between early (CIN1/CIN2) and late (CIN3/CIS) preinvasive OSSN lesions (**Table 3**).We did not notice differences in YAP-1 expression between keratinizing and non-keratinizing SCC (p >.05) or between T1/T2 and T3/T4 stages in invasive cancers (p >.05). However, grade 2 and 3 tumors had significantly higher nucleus YAP-1 overexpression staining intensity than grade 1 tumors (Fisher’s: p = .0078, MC: p = .0489) (**Table 3**).

**Table 3:** Relationship between the Immunostaining intensity-distribution index (IIDI)and the histologic variables of the ocular surface squamous neoplasia.

IIDI of YAP-1 Expression				
	Weakly Positive	Moderately Positive	Strongly Positive	Statistic (I) p- value
OSSN				
Preinvasive	2/5 (40.0)	21/58 (36.2)^#^	39/164 (23.8)	(F) .0076
Invasive	3/5 (60.0)	37/58 (63.8)	125/164 (76.2)	(MC) .1275
Preinvasive				
CIN I/CIN II	0/2 (0.0)	4/21 (19.0)	3/39 (7.7)	(F) .1112
CIN III/CIS	2/2 (100.0)	17/21 (81.0)	36/39 (92.3)	(MC) .3901
Invasive OSSN				
Keratinizing	3/3 (100.0)	34/37 (91.9)	120/125 (96.0)	(F) .1589
Non-Keratinizing	0/3 (0.0)	3/37 (8.1)	5/125 (4.0)	(MC) .4681
Invasive OSSN Grade				
G1	1/3 (33.3)	8/37 (21.6)^#^	11/125 (8.8)	(F) .0078
G2/G3	2/3 (66.7)	29/37 (78.4)	114/125 (91.2)	(MC) .0489
Invasive OSSN Stage				
T1/T2	2/3 (66.7)	19/37 (51.4)	59/125 (47.2)	(F) .0491
T3/T4	1/3 (33.3)	18/37 (48.6)	66/125 (52.8)	(MC) .7267

MC (Monte Carlo), F (Fisher’s exact test).

**Figure 3:**
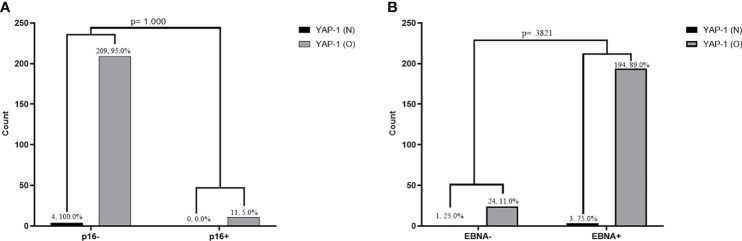
Association between YAP-1 overexpression and P16 expression infection in tumor cells of ocular surface squamous neoplasia **(A)**. Association between YAP-1 overexpression and Epstein Barr virus nuclear antigen positivity in tumor cells of ocular surface squamous neoplasia **(B)**. (YAP-1 N = normal expression, YAP-1 O = over expression).

We found no significant association between YAP-1 overexpression and Epstein-Barr virus infection (EBNA1 IHC) in OSSN (p = .3821; Fisher’s). Similarly, we established no association between YAP-1 overexpression and high-risk human papillomavirus infection (p16 IHC) in OSSN (p = 1.00) (**Figure 3**). We found no association between YAP-1 expression with HIV status (p = .580) (**supplementary Table 2**).

## Discussion

YAP-1’s oncogenic role in the carcinogenesis of several human neoplasms has been studied (55–57) and it has recently been found to drive SCC initiation and progression (25, 58). YAP-1 is highly expressed in SCC from different body sites (59). The current study detected significant upregulation of YAP-1 expression (by immunohistochemistry) in preinvasive and invasive OSSN lesions compared to normal adjacent benign squamous epithelium, and its expression was significantly greater in poorly and moderately differentiated types than in well-differentiated invasive cancer subtypes. To our knowledge, no prior work has evaluated YAP-1 expression in OSSN.

Previous research has found an association between OSSN and prolonged exposure to ultraviolet radiation, immunosuppression such as that caused by HIV/AIDS, Human papillomavirus infection, aging, chronic inflammation, tobacco use, and p53 mutations (1, 49, 60, 60) but no study has considered a role of YAP-1 in OSSN. Given that YAP-1 is highly expressed in SCC from diverse body areas, it is intriguing to evaluate whether YAP-1 expression has a role in the etiology and progression of preinvasive to invasive OSSN. The present analysis found YAP-1 overexpression in preinvasive and invasive SCC arising in the ocular surface, like SCC from other body sites, including the skin, mouth, and esophagus (59).

Previously, in other studies (25, 37, 61, 62), normal control epithelial tissue under normal circumstances expresses YAP-1 in the cytoplasm of the basal cell layer in an inactive state and functions as a sensor of the cellular milieu and is activated in response to cellular stress, such as chronic inflammation or tissue damage, releasing regenerative signals in the basal cell layer stem cells (63, 64).

Our study, however, found that the adjacent normal epithelium within the conjunctiva expressed YAP-1 in the cytoplasm and the nucleus. Cytoplasmic YAP-1 expression was weak in intensity and restricted to the basal keratinocytes, like in the skin, mouth, and esophagus. We also observed nuclear expression of YAP-1 in normal conjunctiva tissue; however, unlike its expression in OSSN, it had a scattered pattern involving less than 25% of the basal keratinocytes and was restricted to the basal and parabasal layer with a weak to moderate staining intensity.

OSSN, however, had a higher basal staining pattern involving more than 80% of the basal cells, with moderate to strong staining intensity and spreading in a parabasal pattern to involve more than 2/3 of the epithelial thickness. Scattered nuclei staining was also observed in other solid tumors, including tumors of the colon, lung, and ovary (65)(Steinhardt et al., 2008). Hence, we propose that scattered nuclear YAP-1 positivity restricted to the basal-parabasal layer is probably progenitor or reparative cells (19, 65) and should be considered normal staining in the ocular surface epithelium. We propose that YAP-1 staining in normal conjunctiva should be studied to determine normal ranges of YAP-1 staining; however, this may be hindered by the inability to recruit normal conjunctiva tissue donors.

YAP-1 protein expression is known to be altered by viral oncogene expression. Recent studies have shown that HPV, PyV, KSHV, Adenovirus, and EBV lead to alteration of the Hippo pathway such that Yap-1 protein accumulates in the nucleus (39–44). In the case of EBV, LMP-1 expression leads to increased protein expression of TAZ, stabilizing nuclear YAP-1 expression in transformed cells (45). Thus YAP-1 is a promising biomarker not only for EBV-related cancers but also for other virally-induced cancers. However, our study did not find any association between YAP-1 overexpression and EBNA-1 or p16 positivity in OSSN; this may necessitate further investigation.

Consistent with our findings, most studies provide compelling evidence that YAP-1 is highly expressed in SCC from various body sites, and it plays a role in the development and progression of this cancer. Several other studies have investigated YAP-1 expression in SCC and its potential as a therapeutic target. It was found that YAP-1 expression was significantly higher in SCC tissues compared to normal tissues in the oral cavity and that high YAP-1 expression was associated with poor prognosis (19, 37, 66, 67). Similarly, they found that YAP-1 was overexpressed in the SCC of the esophagus (24, 64, 68), and of the tongue (25, 69, 70). Others found YAP-1 overexpression in cutaneous SCC and their precursor lesions relative to normal skin (37, 71). Other squamous tumors with significant YAP-1 overexpression relative to the adjacent normal surrounding mucosa include cervical SCC (20) and head and neck squamous cancers (72–74) In light of the association between abnormal YAP-1 expression and tumor formation, it has been hypothesized that inhibiting YAP-1 may prevent the development or progression of squamous cell carcinoma (SCC) (63).

There is evidence that YAP-1 is overexpressed in different types of cancer, including cancers of the breast, pancreatic, colon, lung, ovary, and central nervous system (52, 75–79). It is also worth noting that YAP-1 may be involved in the development of other diseases in addition to cancer. For example, YAP-1 has been implicated in developing cardiovascular disease, liver fibrosis, and other conditions (52, 76–79).

YAP-1 expression may vary depending on the specific subtype of SCC. For example, one study found that YAP-1 expression was significantly higher in keratinizing SCC compared to non-keratinizing SCC of the oral cavity and skin (37). However, we found no difference in YAP-1 expression based on keratinizing *vs*. non-keratinizing, early *vs*. late-stage cancer, preinvasive *vs*. invasive OSSN, and early *vs*. late preinvasive lesions. We found that grade 2 and 3 invasive squamous cancers had significantly higher intensity staining for YAP-1 than grade 1 tumors.

YAP-1 is a transcriptional co-activator that, through dysregulation of the Hippo-YAP pathway, has a role in cancer genesis and progression (80). Many signaling pathways, including the Hippo pathway, regulate its activity. This is accomplished by targeting growth factors and cytokine-producing genes, such as connective tissue growth factor, cysteine-rich angiogenic inducer-61, and tyrosine-protein kinase receptor gene, as well as epithelial-mesenchymal transition, anti-apoptosis, maintenance of stem cells, and cell proliferation, invasion, and metastasis (81). YAP-1 overexpression has been linked to transcription factors that govern morphogenesis, differentiation, proliferation, and apoptosis, including PAX3, SMADs, P73, T-box transcription factor-5, RUNT-related transcription factors, erythroblastic oncogene-B4, and NK2 Homeobox-1 (82). While activated YAP-1 protein frequently accumulates in tumors, YAP-1 and WWTR1 gene mutations are uncommon in human malignancies, including SCCs (56).

While we do not have outcome data for our patients, it is noteworthy that meta-analysis has shown that overexpression of YAP-1 is significantly associated with poorer prognosis and promotes resistance to therapy in patients with various cancers, including SCCs (68, 83), making it a valuable biomarker for predicting prognosis in cancer patients.

*In vitro* studies and scratch wound experiments on SCC cells show that overexpression of YAP-1 in cells with relatively low YAP-1 activity boosted proliferation, decreased apoptosis, and enhanced migration in scratch wound experiments (84, 85). On the contrary, SCC cells with high YAP-1 activity show deceased cell growth, increased apoptosis, and decreased migration with YAP-1 siRNA knockdown experiments (84, 85).Similarly, in orthotopic xenograft trials in which SCC cells were injected into the tongue of nude mice, shRNA suppression of YAP-1/TAZ lowered primary tumor volume and nearly eliminated metastasis formation after 22 days. YAP-1 activity was elevated in an oral SCC cell line selected for resistance to the chemotherapeutic cisplatin relative to the parental line, and inhibiting YAP-1 by siRNA knockdown improved the sensitivity of OSC-19-R cells to cisplatin treatment experiments (59, 84). The above evidence reveals that YAP-1/TAZ plays a crucial role in accelerating the growth, invasion, and metastasis of SCC cancers of various origins. Therefore, targeting YAP-1 may be a potential cancer prevention and treatment strategy.

Finally, it is worth noting that YAP-1 is not the only factor involved in SCC. Other factors, such as p53 and p63, have also been shown to play essential roles in the development and progression of SCC (59, 86). Further research is needed to fully understand the complex interplay between these various factors in SCC, including that arising from the ocular surface (OSSN), and to determine the most effective therapeutic approaches for this cancer. Future studies should prioritize mechanistic research to elucidate the role of YAP-1 in the development of ocular surface squamous neoplasia (OSSN) by examining additional essential factors within the Hippo pathway, such as MST1/2, SAV1, LATs1/2, and TAZ, among others, as this is the main limitation in our study.

## Conclusion

In conclusion, our study detected significant overexpression (upregulation of YAP-1 expression) by immunohistochemistry in preinvasive and invasive OSSN lesions compared to normal adjacent benign squamous epithelium, and its expression was significantly greater in poorly and moderately differentiated than in well-differentiated invasive squamous cancer. YAP-1 overexpression in OSSN but not the adjacent normal epithelium shows that it plays a role in the development and progression of OSSN. Further research is needed to understand the mechanisms by which YAP-1 contributes to OSSN fully and to determine if targeting YAP-1 could be a potential therapeutic approach for SCC.

## Data availability statement

The raw data supporting the conclusions of this article will be made available by the authors, without undue reservation.

## Ethics statement

The studies involving human participants were reviewed and approved by The University of Zambia Biomedical Research Ethics Committee (UNZABREC IRB # 015-05-17), the Zambia National Health Research Authority, the University of Nebraska-Institutional Lincoln’s Review Board (IRB # 20170817442FB), and the Louisiana State University’s Institutional Review Board (IRB # 2252). The patients/participants provided their written informed consent to participate in this study.

## Author contributions

Conceptualization, CW, PA, and PJ. Curation of data, PJ, SS, PA, and FM. Funding acquisition, CW, and PA. Methods, PJ, CW, PA, JW, and TK. Writing Original draft, PJ. All authors contributed to the article and approved the submitted version.
