# Supplementary material for: Yes-associated protein-1 overexpression in ocular surface squamous neoplasia; a potential diagnostic marker and therapeutic target
Source: Front Oncol. 2023 Jul 5;13:1213426. doi: 10.3389/fonc.2023.1213426 (PMC10354641; doi:10.3389/fonc.2023.1213426)
Supplement: Supplementary file 1 [file DataSheet_1.pdf]

Supplementary Table 1: *YAP-1 expression* in combined (preinvasive and invasive) *ocular surface squamous neoplasia (*OSSN).

**Characteristic**	**N = 227**[1]
**Nuclear YAP-1 Final**	
Normal expression	4/227 (1.8)
Overexpression	223/227 (98.2)

Supplementary Table 2: Association between YAP-1 expression with Sociodemographic and Clinicopathology data

Variable		F/T (%); Median [IQR] Range; Mean ±SD	YAP Expression		
			**Normal expression (n=4)**	**Overexpression (n=223)**	**p value**
**Age**	n=227	38 [31-45] 19-76 yrs.	32 (31-46) 19-64 yrs.	38 (31-45) 20-76 yrs.	.720
**Sex**	Female	138/227 (60.8)	2 (50.0)	136 (61.0)	.650
	Male	89/227 (39.2)	2 (50.0)	75 (39.0)	
**HIV status**	Positive	160/227 (70.5)	2 (50.0)	158 (70.9)	.580
	Negative	67/227 (29.5)	2 (50.0)	65 (29.1)	
**HIV long**	n=142	2.0 (0.6 - 5.0) 0.1 - 31.0 yrs.*	3.7 (2.5 - 4.8) 1.3 - 6.0 yrs.*	2.0 (0.6 - 5.0) 0.1 - 31.0 yrs.*	.062
**CD4 Count (cells/mm^3)**	n=144	210.0 [116.0 – 404.0] 4.0 - 1,383.0	757	205 (115.0 - 402.0) 4.0 - 1,383.0	-
**CD4 Count (C)**	<200	69/144 (47.9)	0 (0.0)	69 (48.3)	-
	≥200	75/144 (52.1)	1 (100.0)	74 (51.7)	
**Plasma Viral load (copies/mL)**	n=84	30 [0.0 – 3,992.0] 0.0 – 2.0 x 10^6	8.6 x10^5	30 (0.0 - 2,511.0) 0.0 - 2.0x10^6	-
**Plasma Viral load (C)**	<200	54/84 (64.3)	0 (0.0)	54 (65.1)	-
	≥200	30/84 (35.7)	1 (100.0)	29 (34.9)	
**ART Ever**	Yes	130/160 (81.2)	1 (50.0)	129 (81.6)	-
**ART long**	n=131	2.0 (0.6 -5.0) 0.0 - 20.0 yrs.#	1.3 yrs.#	2.0 (0.6-5.0) 0.0-20.0 yrs.#	-
**Diagnosis**	Preinvasive OSSN	62/227 (27.3)	2 (50.0)	37 (26.9)	.300
	Invasive OSSN	165/227 (72.7)	2 (50.0)	163 (73.1)	
**Preinvasive tumor grade**	CIN-I	1/62 (1.6)	0 (0.0)	1 (0.0)	<.999
	CIN-II	6/62 (9.7)	0 (0.0)	6 (10.0)	
	CIN-III	18/62 (29.0)	0 (0.0)	18 (30.0)	
	CIS	37/62 (59.7)	2 (100.0)	35 (58.3)	
**Preinvasive tumor grade (C)**	CIN I and II	7/62 (11.3)	0 (0.0)	7 (11.7)	>.999
	CIN III and CIS	55/62 (88.7)	2 (100.0)	53 (88.3)	
**Invasive tumor subtype**	CSCC	157/165 (95.2)	2 (100.0)	155 (95.0)	>.999
	BSCC	4/165 (2.4)	0 (0.0)	4 (2.5)	
	SPCC	4/165 (2.4)	0 (0.0)	4 (2.5)	
**Invasive tumor grouped**	Keratinizing	157/165 (95.2)	2 (100.0)	155 (95.1)	>.999
	Non-keratinizing	8/165 (4.8)	0 (0.0)	8 (4.9)	
**Grade of invasive tumor**	G1	20/165 (12.1)	1 (50.0)	19 (11.7)	.350
	G2	133/165 (80.6)	1 (50.0)	132 (81.0)	
	G3	12/165 (7.3)	0 (0.0)	12 (7.4)	
**AJCC Stage**	pT1	40/165 (24.2)	2 (100.0)	38 (23.3)	-
	pT2	40/165 (24.2)	0 (0.0)	40 (24.5)	
	pT3	83/165 (50.3)	0 (0.0)	83 (50.9)	
	pT4	2/165 (1.2)	0 (0.0)	2 (1.2)	
**AJCC Stage (C)**	pT1/pT2	80/165 (48.5)	2 (100.0)	78 (47.9)	.230
	pT3/pT4	85/165 (51.5)	0 (0.0)	85 (52.1)	
**P16 in Tumor**	Present	11 (4.9)	0 (0.0)	11 (5.0)	>.999
**EBNA-1 in Tumor**	Positive	197/222 (88.7)	3 (75.0)	194 (89.0)	0.38

F/T (%): Frequency/total Median (IQR) Range; Mean ±SD; (C): categorized *0-9 months = 0.0 - 0.9 months & 1 = 1 year and above
